# Biomarkers for Alzheimer’s Disease: Where Do We Stand and Where Are We Going?

**DOI:** 10.3390/jpm10040238

**Published:** 2020-11-20

**Authors:** Chiara Villa

**Affiliations:** School of Medicine and Surgery, University of Milano-Bicocca, 20900 Monza, Italy; chiara.villa@unimib.it

Alzheimer’s disease (AD) is an age-related neurodegenerative and progressive disorder representing the most common form of dementia in older adults. AD is clinically characterized by significant cognitive impairments, behavioral changes, sleep disorders, and loss of functional autonomy until the patient becomes completely dependent on the care of family members and healthcare workers [1]. As the population ages worldwide, the number of people suffering from AD is growing rapidly, making this disorder a major public health issue. Actually, the leading biomarkers in clinical practice are directed at the early identification of the two neuropathological hallmarks of AD, namely, amyloid-β (Aβ) plaques and neurofibrillary tangles (NFTs), constituted by hyper-phosphorylated paired helical filaments of the microtubule-associated protein tau. The diagnostic criteria rely on the measures of Aβ, phosphorylated (p-tau), and total tau (t-tau) protein levels in the cerebrospinal fluid (CSF) of patients aided by advanced neuroimaging methods such as magnetic resonance imaging (MRI) and positron emission tomography (PET) [2]. However, the pathological changes silently accumulate in the brain over years or even decades before the onset of symptoms. Therefore, the current challenge is the searching for novel biomarkers to optimize the early diagnosis of AD in the pre-symptomatic stages, essential to start treatments and to propose personalized therapeutic solutions to individual patients.

This Special Issue gathers six original research articles, thirteen literature reviews, one commentary, and one protocol on recent efforts toward the discovery of novel biomarker candidates exploited in different research areas, including biological fluids, genetic/epigenetic factors, pathogens, inflammation, metabolism, nutrition, obesity, or neuropsychological changes (Figure 1). It is not surprising that the most of papers are addressed to review the current knowledge about biomarkers detected in different biological fluids, which are mainly related to pathophysiological processes occurring in AD (e.g., vascular dysfunction, neuroinflammation, and synaptic and neuronal integrity). These reviews largely describe and discuss potential biomarkers detected in CSF or blood as well as in alternative non-invasive body fluids and their possible use in early diagnosis [3,4,5,6,7] or ongoing research protocols on AD [8]. Among them, an emerging role of flotillin as promising biomarker for AD has been proposed by some authors [9]. Moreover, to partially overcome the limitations of biological fluids, advanced brain imaging techniques provide an attractive alternative for the identification of AD-related structural and functional biomarkers [10]. Integrated datasets of multi-faceted AD biomarkers and data-driven analytical methodologies may be involved in the application of the “precision medicine”, aimed to unravel many aspects of AD heterogeneity and to expand the current treatment strategies to help guide more effective diagnosis and clinical management of the disorder [11].

Given the central role of genetics in the development of AD, some authors reviewed emerging candidate genes for familial AD, as well as inherited risk factors, in order to improve the prognostic identification and management of the at-risk individuals. A better knowledge of these genes and their correlated molecular defects will further provide potential targets for the treatment of the disease [12]. One study has reported original results on the association between AD-related polymorphisms and cardiovascular risk factors, which influence the progression to the disorder. Therefore, understanding the molecular mechanisms of this interaction could allow the development of new personalized therapeutic approaches for treating AD [13]. Focusing on the occurrence of behavioral and psychological symptoms of dementia in AD (BPSD), other authors have found an interesting association between *APOE* and *MTHFR* genetic variants and BPSD, expanding the knowledge about the BPSD etiopathogenetic mechanisms, which in turn, leads not only improve the clinical/diagnostic assessment, but also to better definite suitable treatments [14].

As an early event in the pathogenesis of AD, some authors speculated that chronic inflammation should be considered as a potential biomarker in the treatment strategies for AD. Interestingly, inflammation is emerging as the central mechanistic link among diabetes, obesity, and cognitive decline in patients affected by AD. These authors discuss how diabetes and obesity could lead to both systemic and neuro-inflammation, hypothesizing an association with impaired mitochondrial health [15]. Indeed, AD has also been suggested as a metabolic disorder, owing to the fact that some genetic risk factors are key mediators in different metabolic pathways, including glucose, lipid, and energetic metabolism [16]. In this regard, Bell and collaborators demonstrate the strong correlation between fibroblast mitochondrial abnormalities and neuropsychological markers, suggesting the use of fibroblast metabolic assessment as an emergent biomarker of AD [17]. Similarly, another study reports that brain metabolism evaluated by 18F fluorodeoxyglucose (18F-FDG) uptake is moderately related to various neuropsychological tests [18]. Moreover, some authors conceived the “development of metabolic and functional markers of dementia in older people” (ODINO) protocol as an innovative multi-dimensional investigation in which clinical, functional, neuropsychological, and biological parameters are coupled with advanced statistical analyses in order to better identify possible biomarkers that can predict the conversion from mild cognitive impairment (MCI), the prodromal stage of dementia, to AD [19].

Among individuals with MCI, two additional papers reported original results. The randomized cognitive impairment study (CARES) clinical trial demonstrated that targeted nutritional intervention with ω-3FAs, carotenoids, and vitamin E significantly improves the cognitive performances [20]. Other authors showed that an experimental assessment of semantic priming in MCI seems to represent a good paradigm to evaluate subclinical impairment of the semantic system in the early stages of the AD pathology [21]. Finally, an outstanding review discussed how neurophysiological techniques, evaluating mechanisms of synaptic function and brain connectivity, may represent valid biomarkers for screening MCI individuals by the application of artificial intelligence (i.e., learning machine) [22].

Based on studies linking different pathogens with AD and age-related cognitive decline, Naughton and collaborators discuss an interesting role of pathogen-associated biomarkers as a novel tool for evaluating and decreasing AD risk across the population [23].

In conclusion, all articles appearing in this Special Issue cover attractive and current topics of a wide range of biomarkers in the basic research, clinical diagnosis, prognosis, and therapeutic strategies of AD, the most common form of neurodegenerative disorder and a major health challenge with significant social and economic consequences. Early diagnosis entailing the ability to detect AD in asymptomatic patients still remains a big challenge. Therefore, implementing a combination of the aforementioned biomarkers into a diagnostic setting may likely allow the identification of at-risk patients during pre-symptomatic stages necessary to start treatments and to suggest personalized therapeutic strategies.

## Figures and Tables

**Figure 1:**
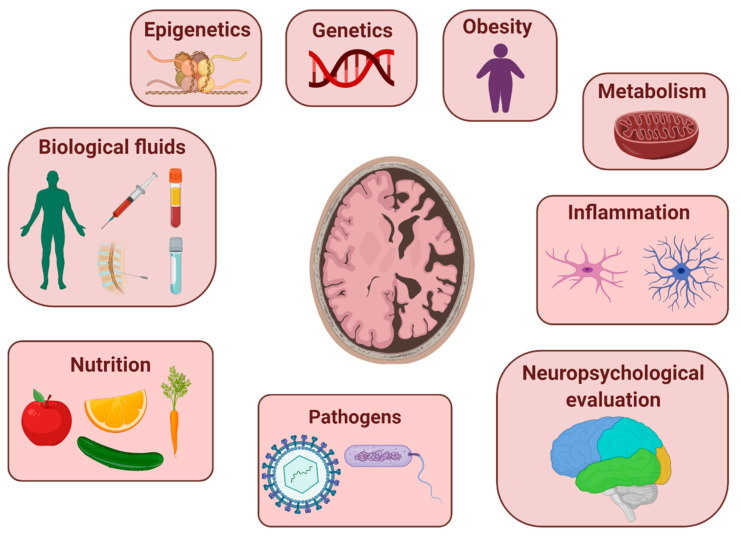
An overview of different research field for exploring potential biomarkers for Alzheimer’s disease. This figure was created with the support of BioRender.com.
